# DNA double-strand break repair gene mutation and the risk of papillary thyroid microcarcinoma: a case–control study

**DOI:** 10.1186/s12935-021-02032-5

**Published:** 2021-07-02

**Authors:** Jiali Qin, Jie Fan, Gang Li, Shanting Liu, Zhensheng Liu, Yao Wu

**Affiliations:** Department of Head and Neck Thyroid Surgery, Affiliated Hospital of Zhengzhou University, Henan Cancer Hospital, Zhengzhou, 450008 China

## Abstract

**Objective:**

To study the relationship between DNA double-strand break (DSB) repair gene mutations and the risk of papillary thyroid microcarcinoma (PTMC).

**Methods:**

One hundred patients with PTMC or benign thyroid nodules (BTNs) at Henan Cancer Hospital were retrospectively analyzed. The DSB repair capacity of peripheral blood T lymphocytes in the two groups was assessed by flow cytometry. Data were compared using Student’s t-test to evaluate the relationship between DSB repair capacity and the risk of PTMC. Factors influencing DSB repair capacity were analyzed by multivariate logistic regression analysis. The relationship between PTMC and DSB repair capacity was analyzed by univariate analysis. Targeted next-generation DNA sequencing was applied to screen and analyze DSB repair genes related to PTMC.

**Results:**

The DSB repair capacity was 31.30% in the PTMC group and 44.40% in the BTN group, with that of the former being significantly lower (*P* < 0.05). Multivariate logistic regression analysis of age, sex, obesity status, radiation and other factors showed that radiation exposure was positively correlated with reduced DSB repair capacity(*OR* = 3.642; 95% *CI* 1.484–8.935, *P* = 0.020). Moreover, univariate analysis showed that a reduction in DSB repair capacity was a risk factor for PTMC**(***OR* = 2.333; 95% *CI* 1.027–5.300, *P* = 0.043).Targeted next-generation DNA sequencing was performed on the DSB repair genes discovered, and those that were mutated in association with PTMC were Rad50 and FANCA; Rad51 mutations were related to BTN.

**Conclusion:**

Radiation exposure is positively associated with induced DSB repair gene mutations, which may cause a reduced capacity for DSB repair and eventually lead to PTMC.

**Supplementary Information:**

The online version contains supplementary material available at 10.1186/s12935-021-02032-5.

## Introduction

The incidence of thyroid cancer has increased in recent years, and it is currently the most common endocrine malignancy. Papillary thyroid carcinoma (PTC) is the most common type of thyroid cancer, accounting for approximately 80–90% [1]. Papillary thyroid microcarcinoma (PTMC) usually refers to PTC with a tumor diameter of less than 1 cm. The degree of malignancy in PTMC is low, and the prognosis is generally good [2]. Nonetheless, the incidence of central lymph node metastasis in PTMC is as high as 37–64%, which is roughly the same as that of conventional papillary thyroid carcinoma [3]. Some patients even experience early recurrence after surgery, distant metastasis in the lungs or bones, and other unfavorable outcomes [4]. Although the pathogenesis and etiology of PTMC remain unclear, possible causes include radiation [5] and obesity [6], among others. For example, radiation is a confirmed exogenous pathogenic factor in thyroid cancer risk, especially in children under 3 years old. Furthermore, there is a significant relationship between radiation and thyroid cancer in children [7]. Abdelaal [8] found that head leakage is an important factor of off-site dose in radiotherapy, especially in head and neck radiotherapy, with harmful effects on healthy tissues around the tumor and leading to the risk of secondary cancer.

The gene repair process is initiated to address DNA damage, and radiation is the most common exogenous factor causing such damage, which can destroy the integrity of the genome. At present, there are at least five DNA damage repair pathways known, namely, base excision repair, nucleotide excision repair, mismatch repair, DSB repair, and direct repair, among which DSB repair is very important [9]. Radiation-induced genomic incompleteness may reduce DSB repair capacity, genetic instability and subsequent cancer [10], though the mechanism of reduced DSB repair capacity remains unclear. Hence, there is an urgent need to identify factors that reduce DSB repair capacity and to analyze the role of candidate molecules in the occurrence and development of PTMC.

Benefiting from advances in molecular biology and precision medicine, the spectroscopy probe designed by Agsalda Garcia [11] emits laser light and captures the scattered light when the tip of the probe contacts tissue; thus, it can be used to provide rapid real-time disease assessment and noninvasive identification of malignant tumors. This technique is of great significance for future diagnosis and prognosis as well as the search for new therapeutic targets. In addition, next-generation sequencing (NGS) technology, such as whole-exome sequencing (WES) and RNA sequencing (RNAseq), involves high-throughput sequencing of genetic materials from different sources, linking various sources of NGS with patient clinical data and constituting a revolutionary change in genomic research [12]. In this study, we aimed to analyze DSB repair genes related to PTMC by NGS technology, screen key and main repair genes, investigate the reasons for reductions in DSB repair capacity, determine the role of these DNA damage repair genes in the occurrence of thyroid cancer, and provide new treatment methods and targets for patients with this cancer.

## Materials and methods

### Study subjects

**Fig. 1:**
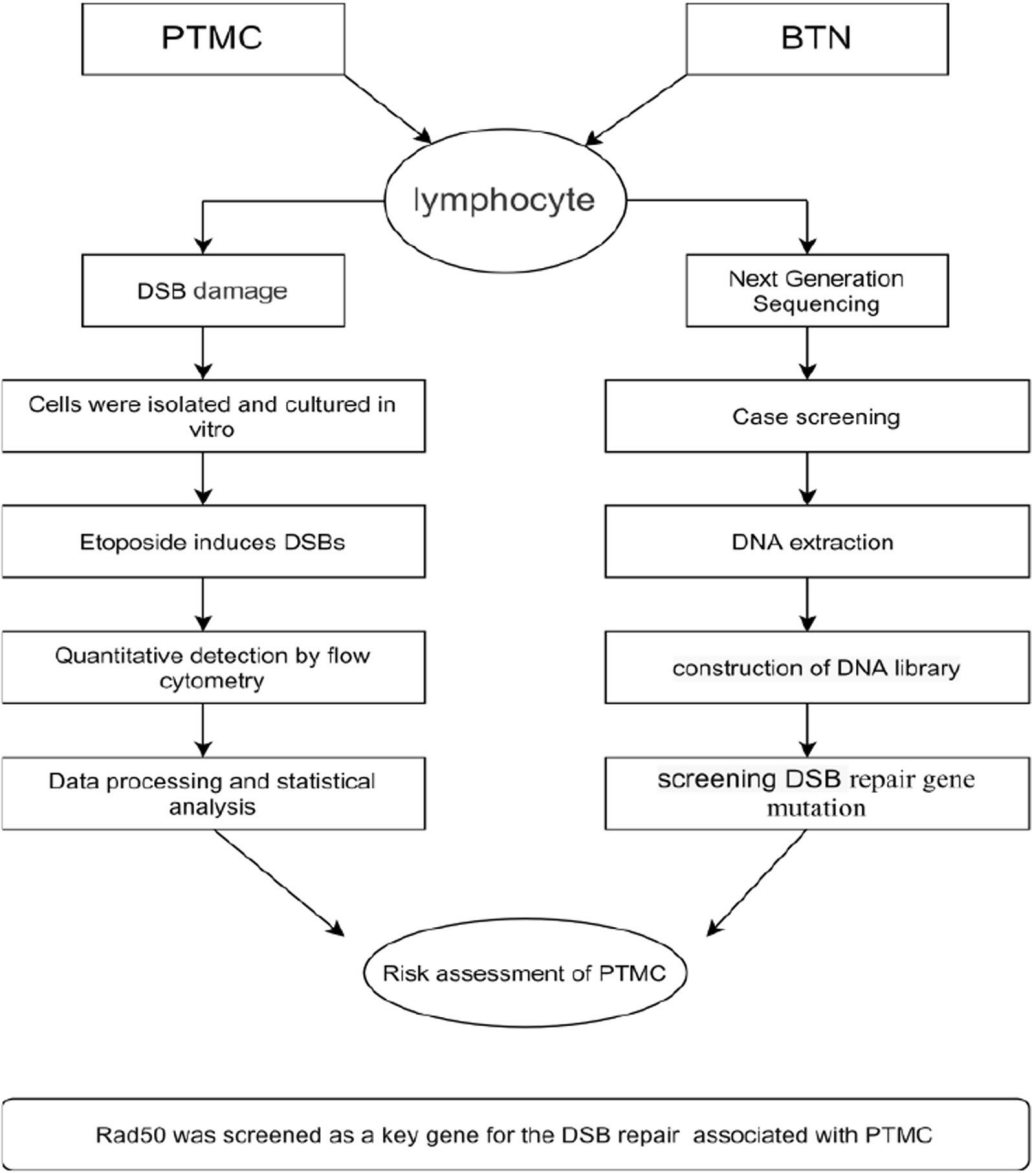
Flowchart of experimental method

The research subjects were 100 thyroid disease patients in our hospital, comprising 19 males and 81 females; the ratio of males to females was 1:4.26. Fourteen patients were ≥ 60 years old, and 86 were younger than 60 years old. There were 48 patients with body mass index (BMI) ≥ 24 and 52 with BMI < 24. The pathological findings were PTMC and BTN in 50 patients each. Of the patients, 9 had a smoking history, whereas 91 did not. Regarding inclusion, all 100 patients with thyroid disease met the following criteria: (1) no history of any other cancer; (2) age 18 years and older; (3) no history of blood transfusion; and (4) no history of immunosuppressive therapy. Radiation exposure was defined as having received more than 3 X-ray examinations before the age of 12 years. Obesity was defined as BMI ≥ 24 kg/m^2^. The subjects were not related. After informed consent was obtained, a questionnaire survey was conducted among eligible subjects, and blood samples were collected. (The flowchart is shown in Fig. 1).

### Collection of blood samples

In this project, 20 ml of complete blood from each subject was collected into two 10 ml anticoagulant tubes with heparin. Lymphocytes (approximately 1 × 10^7^ cells) were isolated from 18 ml of whole blood by gradient centrifugation; the remainder was used for targeted next-generation DNA sequencing. All samples were processed within 8 h and frozen in liquid nitrogen for biomarker determination in batches.

### Treatment of peripheral blood lymphocytes

To detect DSB damage induced by etoposide, T lymphocytes were treated with phytohemagglutinin (PHA). Frozen lymphocytes were thawed rapidly and cultured at 37 °C for 48 h. Each cultured cell (1 × 10^6^ cells were divided into 8 parts) was put into 8 culture tubes of 12 mm × 50 mm: Four of them were DSB injury group (2 etoposide treated for 2 h and 2 drug-free treated tubes as control group);Four of them were DSB repair group (after 2 h of treatment with etoposide, the culture medium with drug was removed and washed with phosphate buffer saline (PBS) once, and then the culture medium without drug was used for 4 h, and two tubes without drug were used for control). Lymphocytes were treated in vitro with an experimental dose (20 μm) of etoposide. The cells were collected, fixed with 1% paraformaldehyde, washed with PBS once, and placed in 70% ethanol at − 20 °C for DSB detection.

### Quantitative detection of DSBs by flow cytometry

Using the γ H2AX assay kit, frozen cells in EP tubes were centrifuged at 5000 BPM; the supernatant was removed, and 1 ml BSA stationary liquid was added and mixed well. After 8 min at room temperature, the cells were centrifuged at 5000 BPM for 5 min, and the supernatant was removed; 100 µl anti-H2AX (Ser139) fluorescent antibody was added and mixed. After 2 h at room temperature, the cells were centrifuged at 5000 BPM for 5 min; the supernatant was removed, and 1 ml BSA was added. The cells were mixed well and centrifuged at 5000 BPM for 5 min; the supernatant was removed, 200 µl RNA inhibitor was added, and 200 μl PBS buffer was added for flow cytometry to count fluorescence-positive and -negative cells.

### Detection of DSB repair genes

FastPure Blood DNA Isolation Mini Kit V2 (NanjingVazyme Company) was used. DSB repair genes such as Rad51, BRCA1, BRIP1, Rad50, FANCA, FANCD2, FANCI, TSC2, MSH3, MSH6, PRSS1, BARD1, and SDHA were sequenced based on second-generation probe capture methods. The sequencing data of the case group and control group were analyzed, and DSB repair genes related to thyroid cancer were screened and verified.

### Statistical analysis

SPSS 22.0 software was used for statistical analysis. Differences in demographic variables (such as age, sex, obesity) between the two groups were analyzed by the χ^2^-test. The difference in etoposide-induced DSB repair capacity level between the PTMC and BTN groups was analyzed by the t-test. DSB repair capacity = 100 × {1 − [percentage of double-strand breaks after 4 h of drug treatment − percentage of double-strand breaks after 4 h of no drug treatment]/[percentage of double-strand breaks after 2 h of drug treatment − percentage of double-strand breaks after 2 h of no drug treatment/[percentage of double-strand breaks after 2 h of drug treatment − percentage of double-strand breaks after 2 h of no drug treatment] [13]. Binary logistic regression analysis was used to examine the relationship between each factor and DSB repair capacity, and the OR value was used to analyze the degree of correlation. Results were considered significant at *P* < 0.05.

## Results

### The general situation

**Fig. 2:**
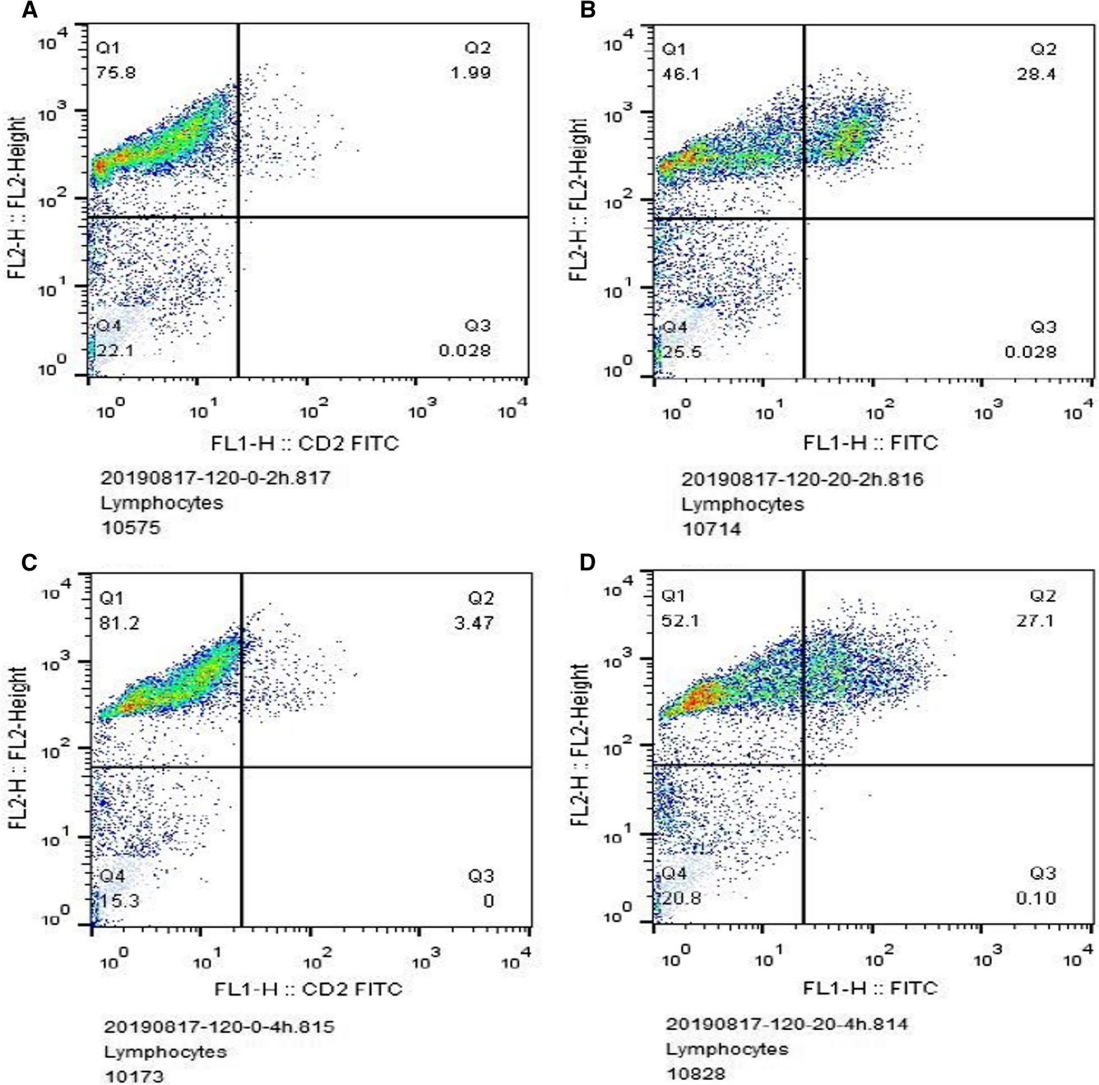
Detection of DSB repair capacity by flow cytometry. **A** Percentage of DSBs treated with etoposide 0 μm for 2 h. **B** Percentage of DSBs treated with etoposide 20 μm for 2 h. **C** Percentage of DSBs treated with etoposide 0 μm for 4 h. **D** Percentage of DSBs treated with etoposide 20 μm for 4 h

There were 50 patients each in the PTMC and BTN groups. No significant differences in age, sex, obesity or smoking history between the two groups were found by the χ^2^-test (*P* > 0.05) (Table 1). DSB repair capacity in peripheral blood T lymphocytes of 100 subjects was measured by flow cytometry. Figure 2A shows that the percentage of DSBs was 1.99 (Q2) after 2 h under normal conditions, Fig. 2B that the percentage of DSBs was 28.4 (Q2) after 2 h of etoposide addition, Fig. 2C that the percentage of DSBs was 3.47 (Q2) after 4 h under normal conditions, and Fig. 2D that the percentage of DSBs was 27.1 (Q2) after 4 h of etoposide addition.

**Table 1:** Characteristics of PTMC group and BTN control group

Variables	PTM (n = 50)	BTN (n = 50)	χ^2^	P
Age (years)			2.990	0.084
≥ 60岁	4	10		
< 60岁	46	40		
Sex			0.065	0.799
Male	10	9		
Female	40	41		
Smoking status			0.122	0.727
Yes	5	4		
No	45	46		
Obesity (BMI ≥ 24)			1.442	0.230
Yes	27	21		
No	23	29

### Relationship between DSB repair capacity and PTMC

**Fig. 3:**
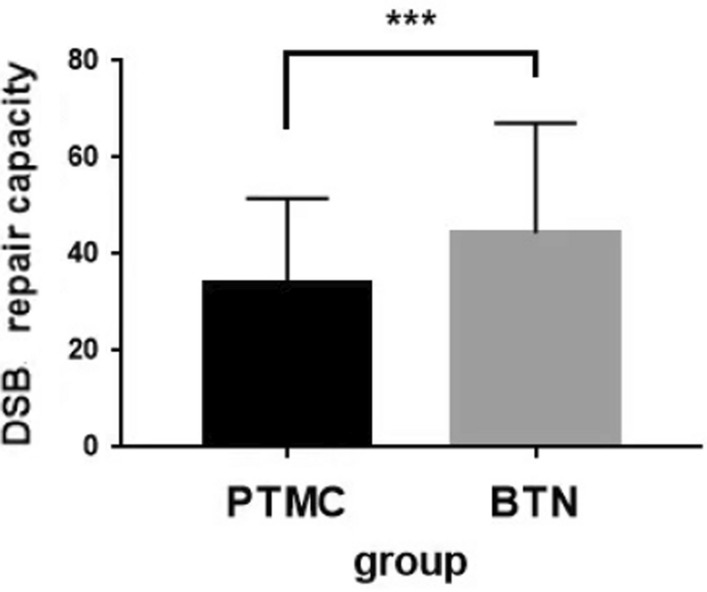
DSB repair capacity of PTMC group and BTN group

Results of the t-test showed that the mean DSB repair capacity in the PTMC group and the BTN control group was 31.30 ± 17.53% and 44.40 ± 22.73%, respectively, which was significantly different (*P* = 0.002) (Fig. 3). Thus, the occurrence of PTMC may be related to a decrease in DSB repair capacity.

### Correlation between various factors and DSB repair capacity analyzed by logistic regression

DSB repair capacity was divided into two groups, high and low, according to the median (37.55%) of the BTN control group. Logistic regression was used to analyze age, sex, obesity, radiation and other factors. It was found that radiation exposure was positively correlated with reduced DSB repair capacity (*OR* = 3.642; 95% CI 1.484–8.935, *P* = 0.005), though there was no significant correlation between age, sex, obesity history and DSB repair capacity (*P* > 0.05) (Table 2).

**Table 2:** Logistic regression analysis for association between DSB repair capacity and various factors

Variables	B	Wald	P	OR	CI	
					**下限**	**上限**
Sex	0.166	0.084	0.772	1.180	0.385	3.620
Age	0.014	0.536	0.464	1.014	0.977	1.052
Radiation exposure	1.292	7.966	0.005	3.642	1.484	8.935
Obesity	0.473	1.199	0.274	1.605	0.688	3.745

### Relationship between DSB repair capacity and PTMC analyzed by logistic regression

Further logistic regression analysis showed that the decrease of DSB repair capacity were positively correlated with the occurrence of PTMC (*OR* = 2.333; 95% *CI* 1.027–5.300, *P* = 0.043).

### Screening DSB repair genes related to PTMC

**Fig. 4:**
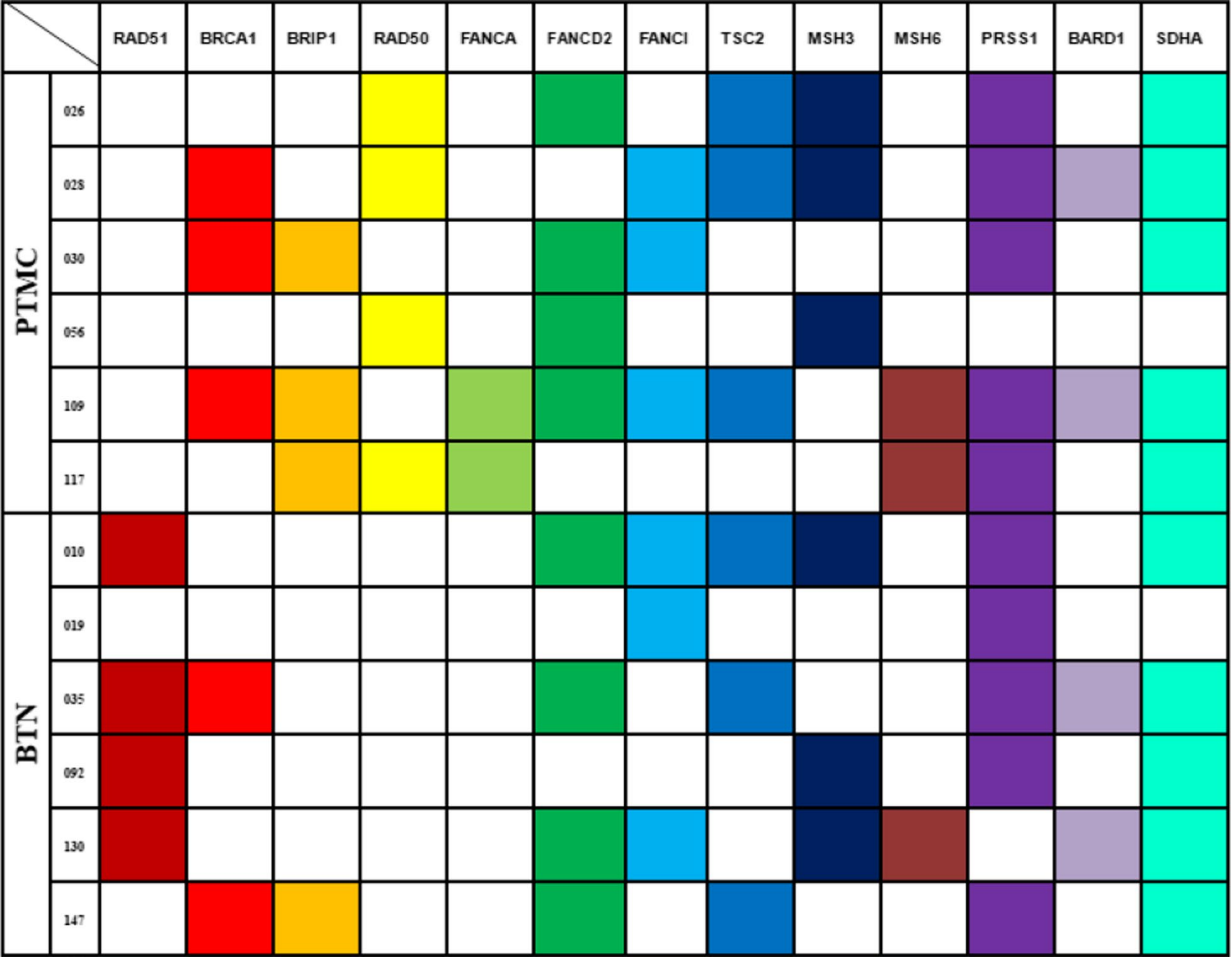
Comparison of DNA damage repair gene mutations between PTMC and BTN

Among 6 patients with PTMC, 4 carried Rad50 gene mutations, 2 had FANCA gene mutations, 1 patient harbored two gene mutations at the same time, and 6 had no Rad51 mutation. With regard to the noncancer control group, Rad50 and FANCA mutations were not found in 6 patients, but Rad51 mutations were found in 4. Although other genes were mutated in the PTMC and BTN groups, no significance was observed (Fig. 4).

## Discussion

DSBs comprise DNA damage, and their accurate repair is essential for the successful maintenance and dissemination of genetic information. There are two main pathways of DSB repair: homologous recombination (HR) and nonhomologous end joining (NHEJ). According to the situation, HR and NHEJ can compete or cooperate to repair DSBs in eukaryotic cells. Defects in both pathways are closely related to disease, including immune deficiency and cancer susceptibility [14, 15]. In addition, the repair process depends on DNA damage repair genes, which generally refer to genes that encode products functionally involved in the recognition and repair of DNA damage. If these genes are mutated, the damaged DNA will not be repaired in a timely and effective manner, or incorrect repair will occur; in general, accumulation of such errors can lead to apoptosis or carcinogenesis. In this study, the DSB repair capacity of the Han Chinese population was assessed; mutations in DSB repair genes were detected, the relationship between DSB repair capacity and PTMC risk was analyzed, and risk factors for a reduction in DSB repair capacity were explored.

The mechanism of many commonly used clinical antitumor drugs, including alkylating agents, DNA-intercalating agents, topoisomerase inhibitors, and antimetabolites, involves different forms of direct or indirect DNA damage. For example, etoposide acts as an inhibitor of topoisomerase II (Topo II), a homodimer that regulates the topological state of DNA by forming ATP-dependent DSBs. As Topo II is an enzyme necessary for cell survival, it has always been an important target of antitumor drugs [16, 17]. Recent studies have shown that DSBs can be detected before DNA replication starts due to etoposide-induced formation of γ H2AX [18]. DSBs induce phosphorylation of serine-139 at the C-terminal conserved region of H2AX. The presence of DSBs can be determined by immunofluorescence detection of γ H2AX with a specific antibody [19]. As the most sensitive biomarker for DSBs, γ H2AX has been widely used in DNA damage research [20], and the ability of γH2AX to detect DNA damage by flow cytometry is reportedly 100 times more sensitive than that of the comet test [21]. In this study, flow cytometry was used to detect DSB repair capacity, and this approach can be applied when studying a large population. The results showed that the DSB repair capacity in the PTMC group was significantly lower than that in the BTN group (*P* < 0.05), indicating that the observed decrease in DSB repair capacity was related to the increased risk of PTMC. Although the scale of this study was relatively small, the data showed that the PTMC patients had relatively low DSB repair capacity compared with the control group.

It is generally recognized that radiation is the most common exogenous factor causing DNA damage and inducing a variety of types of damage, including DSBs, single-strand breaks, base damage, purine- or pyrimidine-deficient sites, glycosyl damage and DNA protein cross-linking, among which DSBs are potentially fatal [22]. DSBs can occur not only through exposure to ionizing radiation but also in the process of DNA replication and transcription. Failure to repair DSBs will lead to the loss of genetic information or cell death, and inaccurate repair will lead to chromosome rearrangement. Our study found that DSB repair capacity was significantly lower in the radiation group than in the nonradiation group. Therefore, we speculate that radiation injury may lead not only to DSBs but also to the mutation of DSB repair genes, resulting in reduced DSB repair capacity, which may be a key factor by which radiation injury causes thyroid cancer.

Adjadj [23] divided damage repair genes into repair genes, cell cycle control genes, kinase-dependent signaling pathway genes, endogenous and exogenous metabolism genes, and hormone metabolism genes according to physiological function. Mutation of a DNA damage repair gene resulting in the reduction of DNA repair capacity will halt DNA replication and transcription; moreover, DNA damage will not be repaired, or abnormal repair will occur, leading to chromosome translocation and gene rearrangement [24]. DSB repair genes include FANCA and Rad50 [25, 26]. By studying 6 PTMC patients and 6 cancer-free controls, we found that mutations in Rad50 and FANCA reduce DSB repair capacity and may lead to the occurrence of thyroid cancer. Although Rad51 gene mutation was found in cancer-free patients, no mutation was detected in the PTMC group, indicating that mutation of the Rad51 gene may reduce the risk of thyroid cancer.

Rad50 is an important repair factor characterized by ATPase motifs at the N- and C-terminal regions, and the sequence between the two regions forms two helical domains adopting the shape of a long curl. Rad50 binds with Mre11 and NBS1 to form the complex Mre11-Rad50-NBS1 (MRN), which recognizes signals and initiates DSB repair [27]. Xu [28] analyzed 80 prostate cancer patients as well as 351 prostate cancer patients from The Cancer Genome Atlas (TCGA) and found that abnormal Rad50 gene expression correlated significantly with the invasive progression and low survival rate of this type of cancer. By examining of the relationship between the MRN complex and colorectal cancer, Situ [29] reported that expression of MRN correlates positively with the prognosis of colorectal cancer and that the MRN complex is a potential biomarker and therapeutic target with important clinical application value. These studies emphasize the relationship between Rad50 and the occurrence and development of cancer. Our study also showed that patients with PTMC are more likely to carry Rad50 gene mutations than are those without cancer, which may be related to the decrease in DNA binding and the tether and nuclease functions of the MRN complex caused by Rad50 mutation, resulting in a decrease in DSB repair capacity.

The Fanconi anemia (FA) pathway is a complex network of functionally diverse proteins that is known for its role in mediating interstrand crosslink (ICL) repair [30]. Palovcak A discovered that in a reconstitution system with purified proteins and various DNA substrates, FANCA, a protein central to FA pathway activation and function, catalyzes Single-Strand Annealing(SSA) and strand exchange of complementary DNA oligonucleotides. This property enables FANCA to directly participate in the SSA pathway of DSB repair in cells [31]. In our study, more FANCA mutations were found in PTMC patients, which may be related to the genomic instability caused by FANCA mutation and the decrease in DSB repair capacity.

In KRAS mutation-driven colorectal cancer, Rad51 has been reported to be transcribed by the KRAS downstream target MYC and to participate in the irradiation-induced DNA damage response and repair [32]. In the present study, more Rad51 mutations were detected in the noncancer population, which may be because Rad51 participates in positive regulation of cell proliferation. In general, abnormal proliferation of local tissue cells under the action of various oncogenic factors is inhibited in the presence of Rad51 mutation, reducing the risk of thyroid cancer. These findings offer new prediction and therapeutic targets for thyroid cancer.

Overall, we believe that the reason why patients with reduced DSB repair capacity are more prone to thyroid cancer may be that DNA repair genes maintain the integrity and stability of the DNA genome by reversing DNA damage. Nevertheless, risk factors such as radiation can lead to genetic variation in DNA repair genes, which will impact the capacity for DNA repair and thus affect the risk of cancer.

## Limitations

Because the cases in this study involved patient and control groups that comprised BTN patients, there was some genetic selection bias. For further study, a healthy control group will be included in our follow-up study. We found that reduced DSB repair capacity is associated with an increased risk of PTMC. However, we cannot exclude the influence of other potential confounding factors (such as viral infection status), which were not controlled in this study. Due to the small sample size, the relationship between DSB repair capacity or related repair gene mutations and the risk of PTMC needs to be confirmed by large-sample studies. In addition, key genes were not explored in this study. In future studies, we will investigate the function of Rad50 in the occurrence and progression of PTMC.

## Conclusion

In summary, this study focused on the role of DSB repair gene mutations in thyroid carcinogenesis and explored risk factors that reduce DSB repair capacity. The results showed that patients with low DSB repair capacity have a significantly increased risk of PTMC. In addition, history of radiation exposure is an important factor for a decrease in DSB repair capacity, which may be related to mutation of DNA damage repair genes as a result of radiation. Overall, we found that low DSB repair capacity may be associated with thyroid cancer. We identified Rad50 and FANCA as the most commonly mutated DNA repair genes. Future studies should verify the functions of these genes experimentally and identify other relevant mutated genes. Further studies should also search for simpler and more effective biomarkers for thyroid cancer risk assessment and provide a new scheme for the clinical treatment of thyroid cancer. An elevated level of clinical vigilance should be implemented for the high-risk population with low DSB repair capacity, as early detection through careful physical examination, B-ultrasound screening and close follow-up can improve prognosis (Additional files 1, 2).

## Supplementary Information

**Additional file 1.** Data of Clinicopathological characteristics of Benign Thyroid Nodules.

**Additional file 2.** Data of Clinicopathological characteristics of Papillary Thyroid Microcarcinoma.

## Data Availability

Data are available from the authors upon reasonable request.

## Abbreviations

DSB: DNA double-strand break
PTMC: Papillary thyroid microcarcinoma
BTN: Benign thyroid nodule
OR: Odds ratio
